# Possibilities, Patience, and Perseverance: A Preliminary Analysis of the Needs and Experiences of Ten Older Adults Regarding Their Use of Digital Health Technology

**DOI:** 10.3390/healthcare11111612

**Published:** 2023-05-31

**Authors:** Janienke Sturm, Angelique Dierick, Marilène Christianen, Marjolijn van Gelder, Eveline Wouters

**Affiliations:** 1 School of HRM and Applied Psychology, Fontys University of Applied Science, P.O. Box 347, 5600 AH Eindhoven, The Netherlands; 2 Department of People and Health Sciences, Fontys University of Applied Sciences, P.O. Box 347, 5600 AH Eindhoven, The Netherlands; angelique.dierick@fontys.nl; 3 Department of Research and Education, Catharina Hospital, P.O. Box 1350, 5602 ZA Eindhoven, The Netherlands; 4 School of Allied Health Professions, Fontys University of Applied Science, P.O. Box 347, 5600 AH Eindhoven, The Netherlands; mjl.christianen@outlook.com (M.C.); m.vangelder@fontys.nl (M.v.G.);; 5 Department of Tranzo, School of Social and Behavioral Sciences, Tilburg University, P.O. Box 90153, 5000 LE Tilburg, The Netherlands

**Keywords:** digital technology, health, healthcare, older persons, qualitative research

## Abstract

The COVID-19 pandemic created the need to use digital health resources (DR), as they sometimes were the only option to receive healthcare or social interaction. The aim of this research is to provide insight into the experiences during the lockdown of older people using DR for health in general and the points of improvement they see. A qualitative study was carried out using semi-structured interviews with older persons by telephone. A total of 10 older adults participated, with a median age of 78 years, the majority having a chronic disease. The most important themes for motivation to use health-related DR were ‘urgency’ and ‘usefulness’. Experiences with DR were related to the themes ‘human contact’ and ‘communication’, which were experienced by respondents as facilitated by DR, and ‘time and energy’, which was two-sided. Additionally, most older persons worried about accessibility of DR by all older persons and the support needed. In conclusion, older persons are convinced of the urgency and the usefulness of digital technology for health and healthcare. Time and energy constraints can be alleviated by using DR on the one hand, but this can also be challenging if older persons are less digitally skilled or lack digital literacy. Good and sustained human support is therefore mandatory.

## 1. Introduction

Today, people are getting older and, at the same time, continue to live independently at home for longer. To enable older adults to continue living at home and support their health and self-management, digital health resources such as monitoring health-related data, medication support, or lifestyle and fall detection monitoring are increasingly used in the healthcare landscape [1]. It is argued that by using digital health resources, the autonomy of older adults can be better preserved, and at the same time, the burden of care will be relieved, while the necessary care is still provided [2]. Because the internet is increasingly being used for health purposes, it is important to implement digital health resources in a way that suits the target group in order to improve and enhance its use. This makes it essential to study the experiences that older people have with using digital health technology and what areas for improvement they see to make better use of these applications [3].

Several studies have investigated the use of eHealth and digital health resources by older people. For instance, Hong and Cho [4] found that older people increasingly use the internet for health purposes. They conducted research into health-related internet use, asking respondents whether they ever used the internet for health information, to order medicines or vitamins, to contact companions, and/or to communicate with healthcare professionals. This study showed that older people mainly use the internet to search for health information, but it was also regularly used to communicate with healthcare professionals. The results further showed that inequality in access to such digital resources is decreasing, but that older people still do not fully exploit all the possibilities digital health resources offer. The authors conclude that especially the oldest group of older adults (75+) often lag behind the younger generations, and therefore make less use of the internet for health purposes [4]. Additionally, in more recent literature, studies showed that older people, for the successful use of mHealth and eHealth, need support [5]. Additionally, it was concluded that people with a lower education and a lower income make less use of the internet to search for health information compared to people with a higher education/income [4,6].

Only a few studies go beyond mere usage of digital health resources and also investigate the experiences of older people with the use of the internet in the context of healthcare. For instance, a Swedish focus group study on the experiences of older people with eHealth showed that older people regularly have a lack of skills and experience with eHealth [7]. This study specifically questioned the search for health information, communication with health professionals, and the monitoring of chronic conditions. The people who did have experience with the usage of such digital applications indicated that their expectations were not always met. They encountered difficulties in accessing important information and sometimes found it too complicated to use. The article also mentions that respondents were afraid that digital resources would lead to costs, both on the individual level and in general healthcare costs, as well as the reduced accessibility to healthcare by older persons because of overuse of the online system by younger persons. Furthermore, some respondents were insecure about their ability to use eHealth in emergency situations [7].

More optimistic views are expressed in the study of Ware et al. [8], who conducted focus group research on the interests, preferences, and concerns of Canadian older adults when it comes to eHealth use. All respondents indicated that they saw possibilities in using eHealth, in particular the use of the internet for communication with doctors and for seeking health information. Many saw eHealth as an opportunity to control their health more easily. The main obstacle encountered in this study were privacy issues. The security and reliability of information on the internet was identified as a concern by the respondents. The respondents also indicated that they needed support in developing skills aimed at assessing the information found [8]. 

This more positive view is also encountered in the study of Ienca et al. [9], who studied the views, needs, and perceptions of community-dwelling older adults regarding the use of digital health technologies for healthy aging. Through in-depth interviews with community-dwelling older people, they found that older people generally had a positive attitude towards digital health technologies, for instance because they facilitate communication with family and formal care givers and help them fulfil their wish to age in place and prolong their permanence at home. However, the participants also had concerns, for instance related to safety, privacy, and lack of human contact [9,10].

From these studies, it can be concluded that in order to maximize the use and effectiveness of these digital health resources, it is important that they are implemented in a way that suits the special needs of older people. Unfortunately, although it has been shown that actively incorporating the needs and perspectives of older people in the design and development of digital health applications will benefit the usability and effectiveness of these applications, the involvement of the target group during the design process is often insufficient [3,9,11].

### 1.1. The COVID-19 Pandemic

In 2019, the COVID-19 pandemic started, which caused an increase in the usage of digital health resources. Due to the pandemic, social distancing became a necessity that made it essential for healthcare providers to seek alternative measures to ensure the continuity of healthcare [12]. Furthermore, to deal with the limitations on social interaction, there was an increase in the use of video calling, social media, and mobile messaging among people [13]. 

This situation also offered a unique context for studying technology and technology acceptance. The unified theory of acceptance and use of technology (UTAUT) describes that the experience of added value and ease of use explains the difference in the acceptance of technology by individuals [14]. These factors are also influenced by external factors, and together they predict the adoption of technology. In the UTAUT, voluntariness of use is important, which entails: “*the degree to which use of the innovation is perceived as being voluntary, or of free will*” [15]. The pandemic created a necessity to use digital health technologies, and as mentioned before, it was sometimes the only option to receive healthcare or social interaction on the short term.

### 1.2. Preceding Research

In May/June 2020, during the first COVID-19 lockdown, the authors of the current article studied the extent to which older adults and people with chronic conditions used digital health resources [16]. Digital questionnaires were distributed among older adults and people with chronic conditions. In this survey, digital health resources were divided on the basis of five topics: health, active exercise, healthy nutrition, social contacts, and leisure time. In this manner, we broadened the concept of health and related the use of digital resources to more than only access to healthcare. This study (125 participants) showed that there was an increase in the use of digital health resources for social purposes compared to before the lockdown. The study also identified a risk that some older people and people with chronic conditions are excluded from these digital resources. Several respondents indicated that they were willing to help think about improvement of digital health resources in future research [16], which was the basis of the current study.

Summarizing, most studies in literature focus on eHealth (aimed at the prevention and treatment of diseases) rather than the broader concept of digital health resources, which may also include the use of information on websites, social technology, et cetera. Furthermore, studies focus more on barriers and support needed to use digital health resources rather than on recommendations to improve access. Finally, especially in the context of COVID-19 and thereafter, it is important that research into the experiences of older adults with digital health resources is continued. The pandemic created an increase in the use of digital health resources, which yielded more experience in the target group. At the same time, the need to use digital health resources created a possible threat to health equality. Therefore, the main question for this research is: 

***How and to what degree have older persons used digital health resources during and after the lockdown and what are their experiences and recommended points of improvement?***

The aim of this research is to provide insight into the experiences of older people (70+) using digital resources for health in general and the points of improvement they see. This article takes a broad perspective towards health, because the pandemic also showed that health is closely linked to, for example, social and emotional wellbeing. 

## 2. Materials and Methods

### 2.1. Health

In this study, we take a broad view on health by using the concept of *positive health* by Huber et al. [17]. Positive health does not primarily focus on the presence or absence of disease to define health but extends this to a broader scope. Positive health focuses on coping with physical, emotional, and social aspects of life and self-direction. There are six dimensions that describe positive health: bodily functions; mental functions and experience; the spiritual dimension; quality of life; social participation; and daily functioning [17].

As a consequence of taking this broad view on health, digital resources for health should therefore also be considered in a broad sense. That is, not only care-related interactions and the action of searching for health information should be seen as digital resources for health, but also digital resources for social contact, leisure, healthy food, and active exercise.

### 2.2. Design

The current study is a qualitative study involving in-depth interviews conducted by telephone. Persons who were over 70 years of age, without cognitive impairments, living independently, and speaking Dutch or English were eligible for this study. Participants of the previous digital survey study who indicated that they were willing to participate and met the inclusion criteria were approached via email, and they were sent an information letter along with an informed consent form. In order to not only include persons who had (independently or with help) filled in the digital survey, each participant was asked to invite people who were possibly less digitally skilled and who would like to participate in the study. In this way, we intended to include respondents varying in digital skills. This resulted in four new respondents who were less digitally skilled, in addition to the respondents from the survey. 

The data was collected through semi-structured interviews. The researchers stopped including participants when no new and relevant topics were discussed in the interviews and participants only repeated the same themes; in other words, when data saturation was reached. The interviews were structured as follows: first, the general socio-demographic information of the respondent, including (chronic) disease and personal health appraisal, was asked about on a 1–10 scale, followed by four topics: exploration of personal views on health, the use of digital health technology, experiences in using these technologies, and possible improvements for digital health resources. For the full topic list, see Appendix A. A total of three pilot interviews were conducted, after which the topic list was adjusted. The interviews took approximately 30–45 min. 

The study was endorsed by the Fontys Ethical Review Board, with approval reference Gelder25052021FCEO. All participants participated voluntarily after giving informed consent. 

### 2.3. Member Check

After the interview, a written summary was sent to each participant, asking them to check the interpretation and pass on any changes if necessary. Most participants recognized themselves in the description of the interview. A total of three respondents had additional remarks to clarify their responses, which were taken into account in the results. 

### 2.4. Data Analysis

The interviews were transcribed verbatim and analysed consecutively. The analysis consisted of open coding and axial coding [18]. The open coding was performed by studying the transcripts and connecting codes to text fragments. Axial coding was conducted to form overarching themes. All transcripts were independently coded by two researchers (MC and MVG). The two researchers discussed their codings. Additionally, all other authors peer-reviewed and discussed the codings.

## 3. Results

### 3.1. Sample Description and Respondents’ Perspective on Health

In total, 10 respondents were interviewed. At this point, data saturation was reached, as described in Section 2.2. Of these respondents, six were from the previously conducted survey and four respondents were newly introduced by these respondents. All participants lived in Zeeland and Noord-Brabant, two provinces in the south of the Netherlands. All participants spoke Dutch as their main language. The median age was 78 years with a range from 71–83. In the former survey we had 125 respondents, of which 57% were female and 43% were male and the median age was 74. A total of three men and seven women were interviewed. Of the interviewees, four were married, one cohabiting, one was in a relationship, three were single, and from one participant, the status of relationship was unknown. All respondents were retired from professional work. Before their retirement, four respondents had worked as a teacher, and other respondents had a diverse background from the IT sector, nurse, director, company owner, psychologist, and exercise consultant (Table 1).

Looking at objective health, the majority of the respondents had one or more chronic conditions. Mainly conditions that were considered as minor inconveniences by the respondents themselves were mentioned, such as high blood pressure or high sugar. Serious conditions such as Parkinson’s disease or heart complaints were also mentioned.

A number of older adults indicated that they did not have a chronic condition, but they actually did have what they considered age-appropriate complaints, such as heart disease, high cholesterol, et cetera. This implies that certain ailments are considered normal on the basis of age.

*R5: “well, that sort of thing comes with age, so that in itself is not a problem”.*

The average health score given by the respondents themselves is 7.7. The answers ranged from 6 to 9, sometimes 10. The score 8 was given most often. The most common reasoning given for the score was that one could still do whatever one wanted. Health was also compared with others’ health, but one’s own perceived physical health was certainly included in the score.

According to the majority of respondents, their definition of health was ‘being able to perform (day to day) activities without too much pain or discomfort’. Both mental and physical health played a role. For the respondents, it was especially important to be able to perform the tasks around home and to be active, appropriate to their age and without too many impairments.

*R4: “uhm yes that I could do certain things like in the house and in the garden and that goes much slower than before and with much shorter periods in succession”.*

Respondents compared themselves to peers when appraising their health, both when it came to chronic conditions and the use of digital resources.

*R4: “on the other hand, I don’t have ailments that other people have”.*

To stay healthy, the respondents made sure that they got enough exercise and stayed active. Eating well was also mentioned often, as well as regular relaxation.

### 3.2. Use of Digital Health Technologies and Motivations for Use

The respondents mentioned that they used digital technology mainly for searching for health-related information and for making appointments with healthcare professionals. Video monitoring with doctors was often mentioned. 

The data analysis revealed two important themes that influence their motivation: urgency and usefulness. 

#### 3.2.1. Urgency

Respondents experienced that nowadays one cannot act without digital technology. They perceived that if one is not able to use it, there will be unsolved problems. This reflects a sense of urgency. 

*R3: “yes, normally yes, you have to. Nowadays you can’t without it … I mean you can’t book a hotel without technology for example”.*

*R7: “… it is preservation”.*

*R8: “…you have to use these things, you cannot do without. However, it is not my favourite thing to do”.*

*R2: “…if you don’t have a mobile phone, it would be difficult. For example the COVID app. ….”*

#### 3.2.2. Usefulness

Often it was mentioned that the degree of using health technology depends on its usefulness. A few respondents realize that they use digital health resources already very often. They compare it with the alternative, which often means they have to go to another city or healthcare centre. They also consider their independency to be an advantage. 

*R2: “People say that one of the motivations for using health technology is that you do not have to drive to the hospital, you do not have to wait or park your care. Which are good reasons. After all, there will be a period in our lives, that we can’t drive a care anymore, because of our age”.*

*R6: “You can find your information on the internet, so you do not have to go to a library. I also read my newspaper on the tablet and I read books on my e-reader. So it is very convenient”.*

*R1: “I think that these kinds of technology are becoming more common. It is cheaper, you can stay at home, it is very efficient, so it will work very well”.*

*R3: “…it is very nice that you are not dependent of a paper or an appointment. You can check the information whenever you like”.*

*R5: “…our medical doctors are located in cities. So we have to drive by car. How easy is it to have an digital consult”.*

### 3.3. Experiences with Digital Technology

Two main themes emerged from the analysis of the interviews with older adults regarding their experience with digital technology: (1) human contact and communication and (2) time and energy. 

#### 3.3.1. Human Contact and Communication

Participants valued the possibility of fast communication that digital technology provides and appreciated the opportunity to remain in touch with their social contacts, especially during times when this was difficult, such as the lockdown as a result of the COVID-19 pandemic. The social contacts included family and friends/acquaintances, but also healthcare and service-related contacts. Another advantage in relation to communication and contact with other persons was the opportunity digital health resources provide to quickly look up information, which also had a positive effect on communication because it supports the participants’ reminiscence.

*R3: “It is a good thing that I can contact my GP by mail and he can reply at once”.*

*R9: “Digital health technology can be helpful in rural areas, or when a person is old, you can arrange assistance in this way”.*

On the other hand, participants also mentioned some disadvantages of digital health technologies and several prerequisites. For instance, some participants mentioned that they experience an overload of information and communication, and several participants also questioned the reliability of information. In addition, most of the respondents mentioned that they sometimes or regularly need support to be able to use digital resources, and that persons who support them need to be patient, as they tend to forget what has been explained. Specifically related to healthcare, it was mentioned that they would rather not communicate about emotional or life-threatening subjects in a digital manner. 

*R5: “As a patient, when you have a life-threatening condition, I think you will need a face to face conversation. Otherwise, there are many things that can be dealt with using digital technology”.*

*R9: “When people are emotional the use of a screen is not helpful”.*

In general, respondents appear to perceive less depth in digital communication as compared to live conversations. At the same time, they experience that in practice there is little continuity in the GPs’ practice, as a result of the increasing number of GPs working part-time, which makes live contacts in their opinion less valuable and the difference with digital communication less prominent. 

*R1: “In the past I had my own general practitioner, and he knew me very well. Nowadays, we have a healthcare centre with several GPs. In this current situation it is easy to use digital devices”.*

In addition, some participants expressed serious concerns for the accessibility of information for persons with low literacy or low digital literacy. Finally, often participants were annoyed and frustrated with organisations that only provided digital services.

*R3: “* *In the past, you had a hospital stay for one or two weeks for some conditions. So it is a good thing to have remote care now. But it is important being mentally okay for receiving care in this way”.*

*R9: “* *I know a person who receives instruction for injecting insulin by face timing with the nurse. So the nurse can check whether he is doing fine. Otherwise the nurse had to visit this person by night, so this is a benefit. When people have more digital skills, this way of caring can be extended”.*

#### 3.3.2. Time and Energy

Most participants value the use of digital technology for seeking out information and accessing care as advantageous. Because they do not need to travel, it saves them time and energy, which is an advantage, especially for older people. 

*R2: “The COPD and cardiology department in a certain hospital already has a lot of digital contact with clients at home. This is increasing also for diabetes care. So you know you don’t have to sit there in a waiting room every time. The corona crisis has certainly accelerated this development”.*

*R5: “In the past I had to drive to the hospital in Zwolle for an ECG, so yes that is a trip that takes half an hour or 45 min before you arrive”.*

*R7: “One of the blessings of the current time is google, you describe something and in no time it provides you with the information. I love it”!*

In contrast with the savings in time and energy described above, some participants also mentioned the time and energy that the use of digital technology costs. For instance, it takes a lot of time and effort to keep up with the technological developments. 

*R8: “Practicing… practicing…, but using digital devices can be exhausting”.*

*R9: “Practicing takes a long time. Repeating, practicing, and remembering”.*

*R4: “Sometimes I hate the devices. Currently I have to use it, but it is not one of my hobbies. Another thing is that my age is an influencing factor. When I do something new, then I should practice every day. Otherwise, my new skills will disappear in a split second”.*

The need for regular updates and replacement of mobile phones and the effort this takes are perceived as time- and energy-consuming. In this context, participants also mentioned the energy and time taken by unclear websites, the ‘jungle of information’ and information loops, frustration when answers could not be found, and the continuous stream of notifications. All this made a simple telephone call much easier compared to using digital health technology. 

*R4: “When I have a complaint, I have to describe it in an e-mail. This takes a lot of time and that annoys me”.*

*R2: “* *I did succeed to make a digital appointment with my general practitioner and I can open some images he sent me. I haven’t tried facetiming with my GP”.*

### 3.4. Points for Improvement

In the interviews, social support, development of digital support, and alternatives for people with low literacy were mentioned as points of improvement. All respondents mentioned the relevance of social support, preferably nearby. Those who give support to older people have to be patient, because training in new digital skills takes a lot of time. One respondent advises to start with an interesting subject or hobby, which can increase motivation to use digital resources. 

*R9: “We have developed a system with “super-users”. We all sit at a large table and one person explains what has to be done and how to use our smart phone; which buttons we have to use. This super-user has digital skills and knows how to give us helpful instructions. The ambiance is very good and it feels like a community”.*

Some respondents have concerns about people who have low literacy or who have cognitive impairments. Companies and organisations have to take that into account and offer alternatives. 

*R5: “Some people don’t use digital resources; they are not able to use them, because they can’t afford it, have low literacy or they think they cannot learn it. For those people there have to be alternatives”.*

## 4. Discussion

This study aimed to give insight into the motivation, current experiences, and considered points of improvement of older people regarding digital health resources. In this study, we used a broad concept of health. We found that two main themes influence the motivation to use of digital technology by older adults, i.e., urgency and usability. The experiences could also be summarized into two main themes, namely human contact and communication on the one hand and time and energy on the other hand. As suggestions for improvement or concern, older adults mentioned social support and care for those who are less (digitally) literate. 

Most of the participants of our study suffered from one or more chronic diseases, but nonetheless, considered themselves as healthy, especially in comparison to other older persons. They defined health as ‘being able to perform (day to day) activities without too much pain or discomfort’, which corresponds to the broad view on health offered by the concept of positive health [19]. 

Participants considered being able to use digital technology as a prerequisite nowadays, without which taking care of one’s life, especially one’s health, is very difficult. Especially during the lockdown and in the presence of no alternatives to contact healthcare professionals, this urgency was felt heavily. At the same time, when improvements and concerns were discussed, the older participants stressed the importance of support for less (digitally) literate peers. This corresponds with literature that underpins the need for and potential of digital healthcare [20], but at the same time, the disparities that emerge as a result [21]. 

At the same time, apart from the urgency and challenges related to disparity, older persons in our study perceived the advantages of digital technology related to healthcare access and health in general, especially for them as older citizens. For them, not needing to travel for visiting medical specialists and being able to easily access all kinds of information was perceived as very useful. This also relates to the experience of participants that digital technology can spare time and energy, both goods that are valuable and tend to be relatively scarcer for older persons, e.g., as a result of chronic disease [22]. Therefore, it is worthwhile to further develop these opportunities.

Although according to the participants, technology can potentially support the lack of time and energy that older persons perceive, the downside of the use of digital technology was also explicitly mentioned by participants: learning how to use digital technology, as well as the frequent updates needed and sometimes not being able to find relevant information, were perceived as an energy drain and time-consuming feature of digital technology. This bears important practical consequences, if one wants to implement health-related digital technology on a larger scale for older adults. As digital technology is considered one of the solutions for the shortage of healthcare professionals and to support aging in place [23,24], it is important to continue to invest in the support of older persons in order to learn how to use digital technology, as well as to invest in improving the user-friendliness of websites and platforms [23]. 

Interestingly, the older participants interviewed for this study considered and discussed the use of technology in relation to changes in healthcare in general. For instance, they perceived changes in the communication with their GP as a result of more part time working and less continuity by one and the same GP, and therefore also better accepted the use of digital technology in communication. At the same time, they stressed the importance of human contact, especially when talking about life events or other themes with high personal impact. The importance of ‘a human in the loop’ was also mentioned for the support they need to learn to use digital technology. Characteristics such as patience, repetition (as they tend to forget), and giving older persons the opportunity to learn at their own pace were considered very important to help people be able to keep up with digital technology and profit from the opportunities it offers. 

### Strengths and Limitations

This study was performed just after the lockdown due to COVID-19. Therefore, older persons not only speculated about the use of digital technology in the context of health, but they could tap into actual experiences. The study not only included digitally skilled older persons, but also purposely sampled persons that were not or less digitally experienced. Therefore, they represent a relatively broad spectre of users. Additionally, their mean age was high, and most of them had chronic diseases. On the other hand, most of our participants were highly educated, and therefore do not represent persons with a lower socio-economic status. The interviews were conducted by telephone. Although the researchers noticed that the respondents felt quite comfortable with the telephone interview, especially in a period after COVID-19 and avoiding the risk of becoming infected, with telephone interviews researchers missed the non-verbal reactions of the participants, making it more difficult to obtain in-depth information. This study used qualitative research methods, including convenience sampling. The aim of these methods is to explore in detail the experiences of older people and to clarify how the use of digital technology works for them in their specific context during the COVID-19 period. The intention of this study was not to test hypotheses. As a consequence, the results of this study cannot be generalized to a larger and different population. Therefore, quantitative research is also needed and recommended. Finally, it is specifically recommended to also study the experiences of older people with a lower socio-economic status. 

Despite its limitations, this study clearly brought to light the challenges and opportunities that need to be overcome when intending to deploy digital technology for the health and healthcare of older community-dwelling citizens. As older persons themselves indicate the urgency and usefulness of digital technology, and therefore, are motivated to use it, the challenge is to support them in a manner that reinforces their motivation and takes away barriers. Referring to the core concepts of the technology acceptance model and its follow ups, this means that usefulness and ease of use should be better balanced in order to enhance intention to use and actual use [25]. For older persons, this means they need good human support, especially when grave health issues are discussed in the context of healthcare, but also, in general when it comes to learning how to deal with digital technology. For older adults, a safe learning environment is necessary, including social support by, for instance, relatives, but also by nearby facilities such as public libraries. Important characteristics of persons who support and train are being patient and understanding that for older persons, repetition may be necessary. Apart from digital skills, guidance in how to find relevant information is also important. This also relates to digital health literacy, a topic that participants were concerned about, as they believe many older persons may lack sufficient skills. 

In former research, it was found that technology acceptance by older adults can be facilitated by the younger generation of grandchildren. Grandchildren are often both skilled and approachable when stimulating older adults to use digital technology [26]. In line with the notion that patience and frequent rehearsal are needed in order to acquire proficiency, learning in a safe context in which students serve as peers for older citizens might be a promising concept for future research. 

## 5. Conclusions

Older persons are convinced about the urgency and the usefulness of technology with respect to health and healthcare. Time and energy constraints can be alleviated as a result of the use of technology on the one hand but can also be challenging for those older persons who are less digitally skilled or who lack digital literacy. The help of other persons, especially in communicating grave (health) issues, stays important. For acquiring digital skills, however, other persons are needed. This support should be given repeatedly and patiently. 

Our findings have implications for the daily practice of digital technology use in healthcare. The use of digital technology can provide good health support in older persons, and as such, provide a good alternative in healthcare. Healthcare providers and policy makers, on the other hand, should be aware that investments need to be made in digital support as older persons need person-centred and repetitive support for the successful use of digital technology.

## Figures and Tables

**Table 1:** Respondents.

	Age	Gender	Relationship Status	Field of Work before Retirement	Health Score (0–10)	Chronic Disease
**R1**	71	Female	Living together	IT sector	8, sometimes 10	Parkinson’s, glaucoma, arthritis
**R2**	79	Female	Married	Nurse	8	None
**R3**	84	Male	Married	Director	8–9	None
**R4**	83	Female	Single	Teacher	6	Heart problems
**R5**	81	Male	Married	Teacher	7	Heart problems
**R6**	76	Female	Unknown	Teacher	Unknown	Visual problems
**R7**	75	Female	Single	Shop owner + administrative worker	7.5	Residual symptoms of cerebral infarction and diabetes
**R8**	75	Male	Married	Psychologist + trainer	9	None
**R9**	82	Female	Relationship	Activity counsellor	8	Auto-immune disease
**R10**	77	Female	Single	Teacher	6	Osteo-arthritis and Meniere’s disease

## Data Availability

The data presented in this study are available on request from the corresponding author.

## Appendix A. Topic List

The following topic list was used as the basis for each semi-structured interview.

**Respondent’s own health**

1. Subjective health: On a scale of 1 to 10, how would you rate your overall health? Please explain your score.
2. Objective health: Do you have a chronic condition and which one? Do you take any medication for this?

**Topic 1: Health**

1. When do you consider yourself healthy? Not being sick, not going to the doctor, eating healthy, exercising?
2. What do you do for your own health?

**Topic 2: Use of digital resources for health**

1. For what health-related purpose do you use the computer, tablet or smartphone?
2. Which digital resources do you use for health (computer, smartphone, etc.)?
3. How often do you use it approximately?
4. Do you feel that you are missing something because you do not use (some) digital resources for health?
5. Why do you choose to use digital resources (or not)?
6. What role does your social environment or your doctor/care provider generally play in this?
7. To what extent do you feel that you have sufficient skills to make effective use of digital resources (if you would like to). What would you like to be able to do even better?
8. What problems do you encounter when using these digital resources?
9. How did you deal with the aspects of digital resources that you find difficult?
10. What would you need to make optimal use of digital resources (help with use, access, etc.)?
11. To what extent do you feel that you have sufficient technical resources or access to an internet connection to enable the use of digital resources (if you would like to)?

**Topic 3: Experiences using digital resources for health**

1. What do you think are the benefits of the digital resources that you use?
2. Have you ever used digital resources for your health in the past? (What is the reason you stopped using these?)
3. To what extent has your use of digital resources changed due to the pandemic?
4. How do you see the future of healthcare with regard to digital resources?
5. What do you think of that?

**Topic 4: Improvements in the use of digital resources for health**

1. There are people who cannot use digital resources. What could be reasons for this?
2. What do you think would be the solution or opportunities to include people who fall outside the boat?
